# Towards Biomimetic Robotic Rehabilitation: Pilot Study of an Upper-Limb Cable-Driven Exoskeleton in Post-Stroke Patients

**DOI:** 10.3390/biomimetics11010011

**Published:** 2025-12-26

**Authors:** Develyn I. S. Bastos, Sergio C. M. Gomes, Eduardo A. F. Dias, Pedro H. F. Ulhoa, Raphaele C. J. S. Gomes, Fabiana D. Marinho, Rafhael M. Andrade

**Affiliations:** 1 Postgraduate Postgraduate Program in Biotechnology, Universidade Federal do Espirito Santo, R. Dióscoro Carneiro Filho, 1-27, Vitória 29047-009, ES, Brazil; 2 Postgraduate Program in Mechanical Engineering, Universidade Federal do Espirito Santo, Av. Fernando Ferrari, 514, Vitória 29075-910, ES, Brazil; 3 Postgraduate Program in Electrical Engineering, Universidade Federal do Espirito Santo, Av. Fernando Ferrari, 514, Vitória 29075-910, ES, Brazil; 4 Department of Occupational Therapy, Universidade Federal do Espirito Santo, R. Dióscoro Carneiro Filho, 1-27, Vitória 29047-009, ES, Brazil

**Keywords:** upper-limb exoskeleton, stroke, neuromotor rehabilitation

## Abstract

Stroke is a leading cause of disability, often resulting in motor, cognitive, and language deficits, with significant impact on upper-limb function. Robotic therapy (RT) has emerged as an effective strategy, providing intensive, repetitive, and adaptable practice to optimize functional recovery. This pilot study aimed to describe and evaluate the effects of robotic rehabilitation as a complement to conventional therapy, using a biomimetic activities-of-daily-living (ADL)-based protocol, on upper-limb function in post-stroke patients. Three participants (aged 30–80 years) undergoing occupational and/or physiotherapy received individualized robotic training with a lightweight cable-driven upper-limb exoskeleton, m-FLEX™, twice a week for ten weeks (30 min per session). Movements were designed to mimic natural upper-limb actions, including elbow flexion-extension, forearm pronation-supination, tripod pinch, and functional tasks such as grasping a cup. Assessments included the Fugl-Meyer (FM) scale, the Functional Independence Measure (FIM), and device satisfaction, performed at baseline, mid-intervention, and post-intervention. Descriptive analysis of the tabulated data revealed improvements in range of motion and functional outcomes. These findings suggest that biomimetic protocol of robotic rehabilitation, when combined with conventional therapy, can enhance motor and functional recovery in post-stroke patients.

## 1. Introduction

Stroke is one of the leading causes of long-term disability worldwide, representing a major public health challenge with profound individual and socioeconomic consequences [1]. According to the World Health Organization [2], approximately 12 million people suffer a stroke each year, and around one-third of survivors experience permanent functional limitations. Among the most affected functions are motor, cognitive, and language abilities, with upper-limb motor impairment being one of the most disabling sequelae [3]. Impairments such as muscle weakness, spasticity, loss of coordination, and reduced range of motion (RoM) compromise the ability to perform activities of daily living (ADLs), directly impacting autonomy, social participation, and quality of life [4]. Despite advances in acute care and rehabilitation strategies, restoring upper-limb function remains a significant challenge in post-stroke recovery [5].

In this context, robotic rehabilitation has emerged as a promising therapeutic approach to complement conventional therapies [6]. Robotic therapy (RT) enables intensive, repetitive, task-specific, and individualized training, that are key factors for promoting neuroplasticity and functional recovery [7]. By integrating mechanical assistance, sensor feedback, and control algorithms, robotic systems can guide or augment voluntary movement, facilitating active participation from the patient [8,9]. In addition, robots allow precise monitoring of performance parameters, supporting objective evaluation and individualized adaptation of therapy. Multiple studies have demonstrated that robotic rehabilitation can improve motor control, strength, and functional independence when combined with conventional therapy, particularly in the upper limb, which requires fine motor skills and coordinated movements [10,11].

In the present pilot study, we implemented a biomimetic, activities-of-daily-living (ADL)-based rehabilitation protocol using the cable-driven m-FLEX™ exoskeleton (Symbios Tecnologias Assistivas LTDA, Vitória, Brazil) to assist upper-limb movements in individuals’ post-stroke. ADL-oriented rehabilitation protocols [12] have been shown to improve independence and functional performance in stroke survivors [12,13]. Unlike conventional robotic training approaches that focus primarily on isolated joint movements, our protocol emphasized a sequence of functional actions, including elbow flexion–extension, forearm pronation–supination, tripod pinch, and cup grasping, to simulate natural tasks such as drinking or bringing an object to the mouth. These coordinated arm-and-hand movements were designed to reflect functional motor demands commonly encountered in daily activities. By combining robotic assistance with conventional therapy based on ADL, the intervention called here biomimetic ADL-based protocol aimed to enhance upper-limb motor recovery, promote functional independence, and provide preliminary evidence on the feasibility and potential benefits of biomimetic ADL-oriented robotic rehabilitation strategies.

The remainder of this paper is structured as follows. Section 2 presents the state of the art in upper-limb robotic rehabilitation, highlighting contributions and research gaps. Section 3 describes the methodology applied in this study, using m-FLEX system, its biomimetic rehabilitation protocol, and the clinical procedures adopted. Section 4 reports the results of the experimental study. Section 5 offers a discussion of the findings considering current evidence, and Section 6 concludes the manuscript with final considerations and perspectives for future developments.

## 2. State of the Art

This section reviews the current literature on upper-limb robotic rehabilitation, focusing on robotic architectures, biomimetic control strategies, and ADL-oriented therapeutic protocols. The goal is to contextualize the contribution of the present work within ongoing efforts to improve functional recovery after stroke.

Over the last two decades, several types of robotic systems have been developed to support upper-limb rehabilitation. These devices can be categorized according to their mechanical structure, actuation principle, and control strategy [14,15]. End-effector robots typically interact with the patient through a single point of contact, guiding the hand or wrist to produce joint movements indirectly [16]. While these systems are generally simpler and easier to fit different users, they may offer less precise control over individual joint movements. Body- or wall-grounded multi DoF robots, on the other hand, are wearable robots or exoskeletons that replicate the anatomical structure of the upper limb, aligning mechanical joints with human joints and allowing more natural, physiological movements [17]. Exoskeletons can provide targeted assistance to specific joints, enabling greater control and personalization of therapy [18,19,20]. Within this second category, cable-driven exoskeletons have attracted increasing attention because they offer several advantages over rigid-link systems. Their lightweight design improves comfort and wearability, reducing the inertia that can interfere with natural movement [21,22]. Cable transmissions allow actuators to be placed away from the moving segments, which contributes to a more compact and ergonomic device. Moreover, cable actuation enables compliant interaction with the user, improving safety and transparency during rehabilitation exercises [23]. An example of this new generation of exoskeletons is the m-FLEX (Symbios Tecnologias Assistivas LTDA, Vitória, Brazil), a cable-driven, lightweight upper-limb orthosis designed to assist movements of the elbow (flexion/extension), forearm (pronation/supination), and hand (tripod grasp). Unlike many conventional robotic systems, the m-FLEX focuses on simplicity of donning and doffing, modularity, and user-centered design, facilitating its integration into clinical practice [22,23,24,25].

While device design plays a critical role in enabling effective therapy, rehabilitation protocols based on high-intensity and repetitive movement training have been shown to be essential in promoting motor relearning after stroke [26,27]. Repetition is a key factor for driving cortical reorganization and functional recovery through experience-dependent neuroplasticity [28]. Previous studies have demonstrated that repetitive task-oriented training with robotic devices can lead to significant improvements in upper-limb function [29,30,31]. For example, studies using exoskeleton-assisted therapy have shown enhanced motor control, increased muscle strength, and improved performance in ADLs after structured training programs [32]. However, one of the challenges in robotic rehabilitation is ensuring that the exercises performed are functionally relevant and meaningful to the patient. Conventional robotic training often focuses on repetitive, isolated joint movements, which may not fully translate to improvements in daily life activities [33]. This has motivated the development of biomimetic rehabilitation protocols, inspired by natural human movements and functional tasks. Biomimetic strategies aim to replicate the kinematics and dynamics of daily activities, engaging multiple joints and muscle groups in coordinated actions [34]. This approach can promote more natural motor patterns and increase patient motivation and adherence by incorporating purposeful and familiar tasks. A biomimetic ADL-based protocol involves designing rehabilitation exercises that closely mimic functional movements used in everyday life, such as reaching, grasping, lifting, and manipulating objects. By integrating these natural motor patterns into therapy, patients are exposed to task-specific practice that is directly relevant to their daily routines [35]. Previous research suggests that ADL-based robotic rehabilitation can enhance functional transfer, improve independence, and strengthen neural pathways associated with voluntary control [36,37]. Furthermore, these protocols can be adapted to different levels of impairment, allowing progressive challenge and individualized goals.

Although substantial progress has been made in robotic rehabilitation, current evidence shows that many systems still emphasize isolated joint training and lack protocols that replicate coordinated, functional tasks relevant to daily living [16,34]. To address this gap, the present study proposes a pilot investigation using the cable-driven m-FLEX exoskeleton combined with a structured biomimetic ADL-oriented rehabilitation protocol. This approach aims to evaluate the feasibility and potential therapeutic benefits of integrating functionally meaningful, multi-joint movements into robotic training. By examining clinical outcomes across multiple time points, the study seeks to contribute evidence toward more naturalistic and patient-centered robotic rehabilitation strategies, ultimately supporting improved functional recovery and independence after stroke.

## 3. Materials and Methods

### 3.1. The m-FLEX™

**Figure 1:**
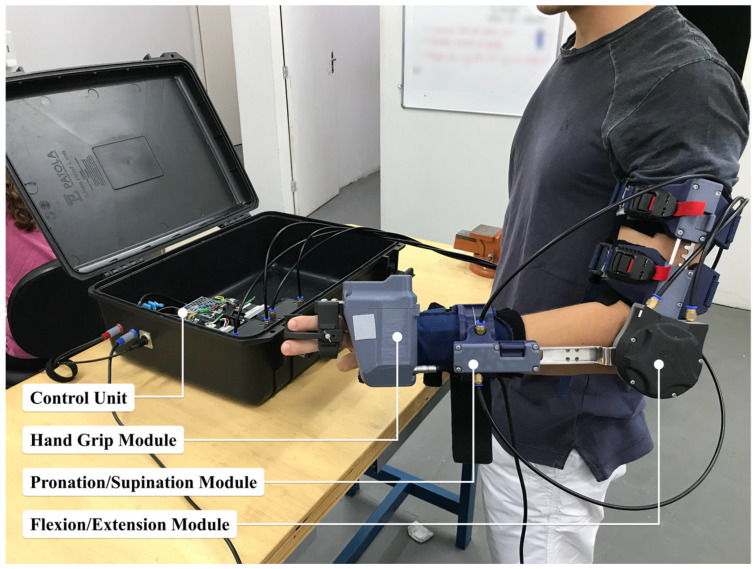
The m-FLEX™ is a cable-driven upper-limb exoskeleton intended for post-stroke rehabilitation, featuring a portable control unit and three functional modules: a hand module for tripod pinch, an elbow flexion–extension unit, and a forearm pronation–supination mechanism.

The m-FLEX™ (Symbios Tecnologias Assistivas LTDA, Brazil) [22,23,24,25], illustrated in Figure 1, is a lightweight upper-limb exoskeleton with three degrees of freedom (DoF), specifically designed to support elbow flexion–extension, forearm pronation–supination, and tripod pinch grasp movements. Its modular architecture allows the optional integration of a hand module, providing flexibility to adapt the device to different rehabilitation needs.

The actuation system uses Bowden cable transmissions, which relocate both the gearmotors and the control electronics to an external portable unit. This configuration reduces inertia in the distal segments, improving user comfort and movement transparency. The transmission employs 1.5 mm stainless steel cables (7 × 19 strand) routed through PTFE-lined conduits to minimize friction losses. Thanks to this arrangement, the entire wearable structure weighs approximately 900 g.

The system consists of four main parts: (1) the portable control unit containing the actuators and electronics, (2) the wearable exoskeleton structure, and (3) interchangeable right- and left-hand modules. The ambidextrous design can be adjusted to different arm and forearm lengths. Proximal attachment is achieved through magnetic fasteners combined with adjustable straps, while distal fixation uses a wrist orthosis equipped with a spring-assisted quick-release mechanism. To ensure user safety, the exoskeleton includes adjustable elbow angle limiters in 15° increments, as well as pronation–supination constraints ranging from −75° to +75°. A summary of its kinematic specifications is presented in Table 1.

For control, the system provides a Python-based software development kit (SDK), version 2.1, that enables high-level command of trajectories, velocity and torque limits, and acquisition of sensor signals (such as encoders and voltages), in addition to built-in safety functions. The communication interface supports bidirectional data transfer and control loops at frequencies up to 100 Hz.

### 3.2. Experimental Protocol

Three volunteers who had experienced a stroke within the previous year (two men and one woman), representing the acute and subacute stages of the disease, were recruited for this study.

Inclusion criteria: individuals aged 18 years or older, of both sexes, with a stroke occurring less than one year prior to recruitment; presenting hemiplegia and motor and/or sensory neurological impairments in the upper limb; exhibiting significant limitations in daily living activities; currently undergoing occupational therapy and/or physiotherapy; and with preserved perceptual-cognitive function.

**Figure 2:**
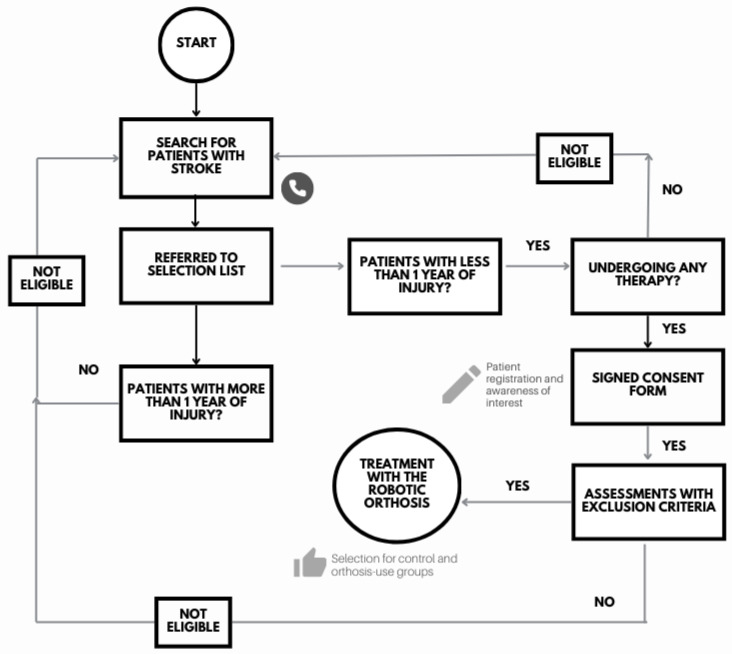
Flowchart of patient recruitment and eligibility assessment. The study began with patient screening in hospitals and rehabilitation clinics. Participants were then evaluated according to the inclusion and exclusion criteria. Eligible patients were enrolled in the study and followed the established experimental protocol.

Exclusion criteria: individuals in the chronic phase of stroke, with cognitive impairments resulting from the stroke or pre-existing conditions (scoring below the minimum threshold on the Mini-Mental State Examination), or those not receiving occupational and/or physical rehabilitation. The participant selection flowchart is shown in Figure 2.

The initial sample consisted of 22 participants. Several factors prevented some individuals from taking part in the study, including scoring below the minimum threshold on the Mini-Mental State Examination, lack of interest, transportation difficulties, stroke onset longer than one year, absence of ongoing rehabilitation treatment, or already being able to perform the movements supported by the orthosis. Consequently, only three volunteers (13.6% of the total sample) with a diagnosis of stroke in the acute or subacute phase were enrolled in the study.

Two of the participants experienced strokes in the left hemisphere, leading to impairment of the right side of the body as a result of middle cerebral artery involvement. Both exhibited expressive or mixed aphasia, hemiplegia, and spasticity. The third participant sustained a right hemisphere stroke that affected the left side of the body; while initially showing no signs of spasticity, this symptom developed later.

**Figure 3:**
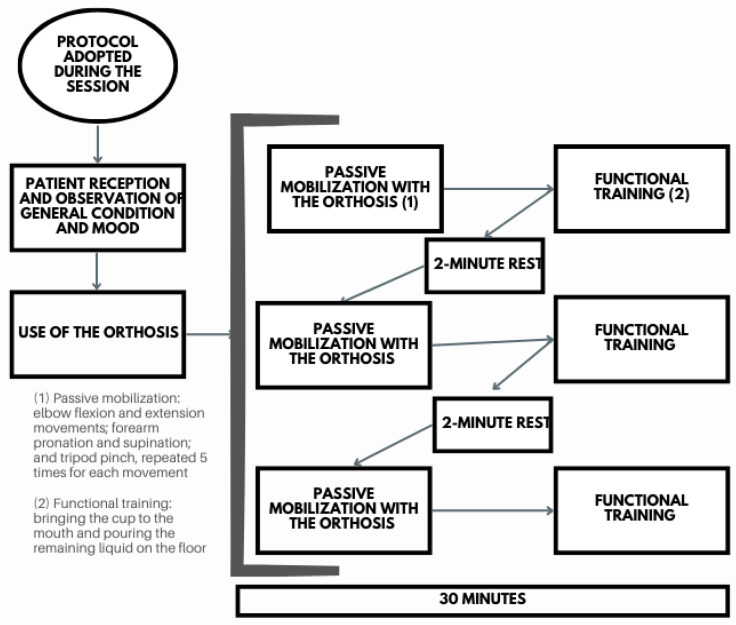
Biomimetic activities-of-daily-living (ADL)-based training protocol. Each rehabilitation session consisted of three sets of five repetitions of elbow flexion–extension, forearm pronation–supination, and tripod pinch, with rest intervals of approximately two minutes. At the end of each set, a functional task was performed in which participants used the orthosis to grasp a cup and bring it to their mouth, involving elbow flexion up to 90°, forearm pronation, and a tripod pinch to hold the cup.

Following admission and baseline assessment, participants underwent 20 robotic rehabilitation sessions over 10 weeks, with two sessions per week and a duration of 30 min each. Each session included three sets of five repetitions of elbow flexion–extension, forearm pronation–supination, and tripod pinch movements, with approximately two minutes of rest between sets (Figure 3). At the end of each set, participants performed a biomimetic functional task with the exoskeleton, replicating a natural activity of daily living (ADL): grasping and bringing a cup to the mouth. This task required a coordinated sequence of movements, including elbow flexion up to 90°, forearm pronation, tripod pinch grasp to secure the cup, continued elbow flexion to bring it to the mouth, followed by elbow extension, forearm supination, and tripod pinch release to place the cup down. By mimicking familiar and meaningful motor action, the protocol aimed to reinforce task-oriented motor learning and promote functional recovery.

Participants were evaluated at three time points: baseline (1st session), mid-intervention (10th session), and post-intervention (20th session), using the Functional Independence Measure (FIM) and Fugl-Meyer Assessment (FMA) scales.

To ensure full transparency and mitigate potential bias related to the development of the m-FLEX™, all clinical procedures were conducted by an independent rehabilitation team. The clinical assessment of participants, the delivery and supervision of the training sessions, and the administration of all functional scales were performed exclusively by licensed therapists who were not involved in the design, development, or technical implementation of the device. The training exercises followed a standardized biomimetic ADL-based protocol, including predefined sequences of reaching, grasping, lifting, and forearm rotation tasks, ensuring consistency across participants. During each session, therapists actively monitored performance, provided verbal and physical cues as appropriate, and adjusted the level of passive and active assistance delivered by the m-FLEX™ to maximize voluntary effort while maintaining safety. Concurrent conventional therapy was maintained according to each participant’s clinical routine, and therapists documented its frequency and content to account for potential confounding effects in the interpretation of outcomes.

The experiments involving post-stroke patients were approved by the Research Ethics Committee of the Universidade Federal do Espírito Santo (CAAE: 41368820.3.0000.5542). All participants were informed about the study procedures and provided written informed consent prior to participation.

## 4. Results

This section presents the results of the three post-stroke patients at baseline (1st session), mid-intervention (10th session), and post-intervention (20th session) of the biomimetic rehabilitation sessions, which involved a biomimetic functional task with the exoskeleton, replicating a natural activity of daily living (ADL), based on the FIM and FMA scales.

**Figure 4:**
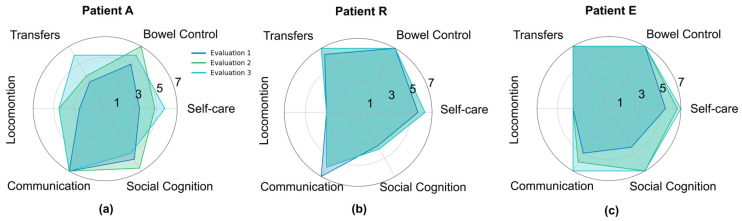

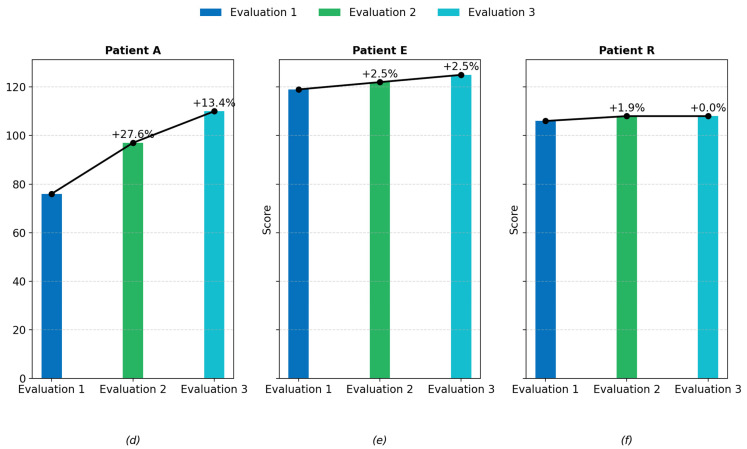
Results for the Functional Independence Measure (FIM) scale for each patient at baseline (Evaluation 1: 1st session), mid-intervention (Evaluation 2: 10th session), and post-intervention (Evaluation 3: 20th session). Results of each category of FIM scale (Self-Care, Bowel Control, Movement, Locomotion, Communication and Social Cognition) for patient A (**a**), patient R (**b**) and patient E (**c**). The final scores of FIM scale of patient A (**d**), patient E (**e**) and patient R (**f**).

The results obtained with the FIM scale are presented in Figure 4. Based on the categories and subgroups of the FIM, it was possible to classify participants according to their level of dependence or independence from the scores obtained. Two participants completed the assessment independently, while one required assistance from the primary caregiver. The mean scores across categories ranged from 1 to 7, indicating varying levels of functional independence among the participants. All individuals showed improvement in their category classification throughout the biomimetic rehabilitation sessions, as illustrated in Figure 4a–c.

The Self-Care category was assessed through the following subgroups: eating, personal hygiene, bathing, dressing upper body, dressing lower body, and toileting. In the “Eating” subgroup, participants demonstrated, on average, modified independence: two participants achieved modified independence, and one achieved complete independence. In “Bathing,” participants initially required supervision or setup, but improvements were observed over time, with two participants reaching modified independence and one requiring minimal assistance. Both “Personal Hygiene” and “Dressing Upper Body” indicated the need for supervision or setup for all participants. For “Dressing Lower Body,” two participants were fully independent, while one required minimal assistance.

The Bowel Control category, which includes urine and feces control, revealed that one participant had modified independence, while the others were completely independent in both subgroups.

The Transfers category included bed/wheelchair, toilet, and tub/shower transfers. In the “Bed and Wheelchair” subgroup, two participants were fully independent, and one required supervision or setup. In contrast, in the “Tub/Shower” subgroup, two participants achieved full independence and one required minimal assistance, suggesting difficulties in this task likely related to balance or motor limitations.

The Locomotion category was assessed through walking/wheelchair use and stairs. One participant achieved total independence in both walking and wheelchair mobility, another maintained modified independence, and the third required minimal assistance. In the “Stairs” subgroup, one participant remained fully independent, while the other two showed improvement but still required assistance.

The Communication category included comprehension and expression. All participants demonstrated full independence in comprehension. However, expression varied considerably: one participant required maximal assistance, another required supervision, and the third was fully independent.

The Social Cognition category included social interaction, problem solving, and memory. In “Social Interaction,” one participant showed modified independence, one required supervision, and one required maximal assistance. In “Problem Solving”, only one participant was fully independent, another required minimal assistance, and the third needed maximal assistance, indicating cognitive and executive function challenges. In “Memory,” one participant was fully independent, one maintained modified independence, and one required supervision or setup.

In summary, the most impaired areas included Expression, Problem Solving, and Social Interaction, suggesting possible cognitive and language deficits. Conversely, the highest scores were observed in Memory, Eating, Lower Body Dressing, Bowel and Bladder Control, Transfers, Gait, and Comprehension, reflecting greater functional independence, either modified or total. These results also indicate cognitive improvements related to language comprehension, mobility, and the use of compensatory and adaptive strategies for performing ADLs. Additionally, Transfers and Locomotion showed notable improvement, although some challenges remained, particularly in the “Stairs” and “Tub/Shower” subgroups.

Figure 4d–f show the final scores for each participant. All patients demonstrated functional improvement according to the FIM scale. Patient A improved from 76 at baseline to 110 at post-intervention, indicating a transition from minimal assistance to modified independence. Similarly, Patient E increased from 119 to 125, reinforcing their progression within the modified independence category. Patient R showed a modest increase of 2 points (106 to 108), which was nevertheless sufficient to change their classification from supervision/setup to modified independence.

**Figure 5:**
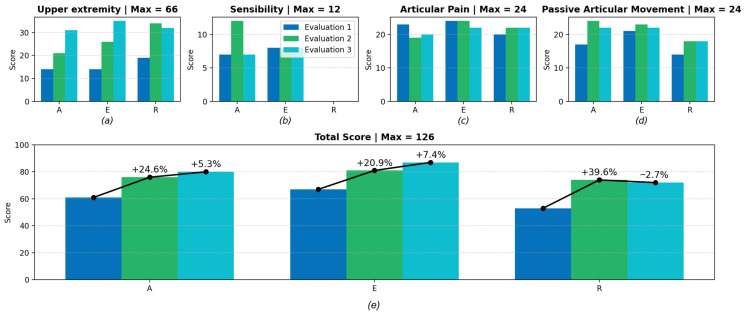
Results for the Fugl-Meyer Evaluation for each patient (A, E and R) during the rehabilitation period, considering 3 evaluations during this period at baseline (Evaluation 1: 1st session), mid-intervention (Evaluation 2: 10th session), and post-intervention (Evaluation 3: 20th session). (**a**) Upper extremity category. (**b**) Sensibility category. (**c**) Articular pain category. (**d**) Passive articular movement category. (**e**) Final punctuation.

Fugl-Meyer Assessment (FMA) for upper-limb sensorimotor function was used to investigate the initial physical performance of the participants, quantify their progress at each assessment stage, and verify the functional gains achieved through robotic rehabilitation combined with conventional therapy. The findings are presented in Figure 5.

In the “Upper Extremity” domain (Figure 5a), all three participants (100%) presented with severe motor impairment at baseline (1st session). After the intervention period (20th session), one participant improved to marked motor impairment, while the other two remained classified as severe. Regarding the “Sensibility” domain, shown in Figure 5b, one participant exhibited absence of sensation, while the other two showed hypoesthesia. These results remained unchanged after the intervention, suggesting persistent tactile and proprioceptive deficits that may hinder functional recovery. In the “Articular Pain” category (Figure 5c), improvements were observed in all movements across participants. Initially, participants reported pain in the shoulder during abduction, internal and external rotation, forearm supination, and finger flexion. Following the intervention, pain during passive movement was reduced in most joints, except for shoulder abduction. In the results presented in Figure 5d, regarding the “Passive Articular Movement” domain, all participants initially demonstrated reduced range of motion in shoulder flexion, abduction, internal and external rotation, forearm pronation and supination, wrist flexion and extension, and finger flexion and extension. After completing the intervention, shoulder movements (abduction, internal rotation, and external rotation) remained limited for all participants, revealing a strong relationship between restricted passive range of motion and persistent joint pain.

The total FMA score for upper-limb sensorimotor function can reach a maximum of 126 points. As depicted in Figure 5d, when comparing the first and last assessments, one participant improved from 61 to 80 points, gaining 19 points (31.14%), with progress in the upper-extremity and passive joint movement domains. This participant remained classified as having severe motor impairment, reduced sensation, some joint pain, and limited joint mobility. Another participant improved from 67 to 87 points, a 20-point gain (29.85%), progressing from severe to marked motor impairment, while maintaining reduced sensation and some joint pain with limited mobility. The third participant improved from 53 to 72 points, gaining 19 points (35.84%), showing better performance in the upper-extremity, pain, and passive movement domains, but still presenting severe motor impairment, reduced sensation, and limited joint mobility. Overall, these results demonstrate measurable functional improvements over the ten-week intervention period, supporting the potential benefits of combining occupational/physical therapy with robotic-assisted rehabilitation using the biomimetic activities-of-daily-living (ADL)-based protocol.

## 5. Discussion

Stroke remains one of the leading causes of long-term disability, frequently resulting in motor, sensory, and cognitive impairments that compromise upper-limb function [3]. In this context, robotic rehabilitation has gained increasing attention as a complementary approach to conventional therapy, providing high-intensity, repetitive, and task-oriented practice that promotes neural reorganization and functional recovery [7]. The present pilot study investigated the feasibility and therapeutic effects of a biomimetic activities-of-daily-living (ADL)-based protocol using a lightweight, cable-driven upper-limb exoskeleton (m-FLEX™) in post-stroke patients undergoing occupational and/or physical therapy.

In the Fugl-Meyer Assessment (FMA), all participants initially presented severe motor impairment. After the intervention, one improved to marked impairment, while two showed meaningful numerical gains despite maintaining the same classification. These results suggest that continued robotic-assisted therapy could further enhance recovery, particularly when delivered at higher intensity and during acute or subacute phases, when neuroplasticity is more responsive. Similar findings were reported by Pollock et al. [5] and French et al. [28], who concluded that intensive, repetitive, and motor learning–based training significantly improves motor outcomes and cortical reorganization after stroke.

Pain in the affected limb, especially shoulder pain, was identified as a limiting factor for motor recovery. Participants reported pain during abduction and rotation movements, consistent with findings by Cacho et al. [38] and Niessen et al. [39], who associated hemiplegic shoulder pain with glenohumeral subluxation, soft-tissue trauma, and proximal muscle spasticity, all of which restrict range of motion and delay rehabilitation progress.

No significant improvements were observed in the sensation domain, indicating persistent tactile and proprioceptive deficits. This limitation likely hindered faster motor recovery, as proprioceptive feedback is essential for regulating muscle force and spatial awareness of limb position. These findings align with studies by Bolognini et al. [40] and Smania et al. [41], which demonstrated that sensory deficits, particularly in proprioception, negatively affect motor learning and functional outcomes after stroke.

Functional independence, assessed by the Functional Independence Measure (FIM), revealed greater dependence on cognitive and self-care tasks such as problem-solving, bathing, and dressing, consistent with previous studies [42]. Nevertheless, improvements were observed in several functional domains, with participants progressing from moderate dependence to modified independence. The correlation between FMA and FIM scores highlights the interdependence between motor and functional recovery, as also reported by Rissetti et al. [43], who demonstrated that motor components are strong predictors of overall functional performance in post-stroke individuals.

The timing of the intervention may have played a critical role in these outcomes. Early and combined approaches, merging robotic and conventional therapies, can potentiate neuroplastic responses, as evidenced by Zeiler and Krakauer [44], who showed that early-phase rehabilitation, coupled with external stimulation, enhances cortical reorganization and functional gains. This supports the integration of robotic-assisted therapy into early rehabilitation programs to maximize recovery potential.

The biomimetic ADL-based protocol adopted in this study emphasized natural, goal-directed movements such as reaching and grasping a cup, aiming to replicate meaningful daily activities. This design fostered patient engagement and motivation, aligning with the concept of client-centered therapy. According to Kennedy and Davis [45], patient engagement and active participation are essential determinants of long-term adherence and rehabilitation success. Encouraging patients to continue specific functional exercises at home, such as the cup-reaching task, proved effective in reinforcing learned motor patterns beyond the clinical environment.

Robotic therapy offers distinct advantages for such biomimetic interventions, including precise control of movement parameters, adaptability to patient performance, and quantifiable feedback. These features facilitate repetitive, intensive, and individualized training, which are key factors for promoting motor relearning and functional recovery [7]. The m-FLEX™ exoskeleton’s cable-driven design provided comfortable, lightweight assistance with minimal mechanical impedance, supporting both safety and compliance, as highlighted by Manna and Dubey [46].

Participant satisfaction was notably high (mean score 4.67/5), reflecting both comfort and acceptance of the device. The adjustability and ease of use of the exoskeleton likely contributed to this outcome, reinforcing the importance of ergonomic design and proper user training. High acceptance is crucial for therapy adherence and for transitioning from clinic-based to home-based rehabilitation routines.

Overall, the results of this pilot study suggest that a biomimetic ADL-based robotic protocol, when integrated with conventional therapy, may support improvements in upper-limb motor performance and functional independence in individuals’ post-stroke. However, these findings must be interpreted with caution due to some important limitations. First, the small sample size (*n* = 3), the absence of a control group, and the concurrent application of conventional therapy prevent any robust conclusions regarding the efficacy of the intervention. In addition, the training intensity and total number of sessions were relatively low, which may have constrained the magnitude and stability of functional gains. The outcome measures employed, primarily FMA-UE and FIM, capture global aspects of motor function and independence but do not isolate the specific contribution of the robotic component, nor do they fully reflect sensor-based quantitative parameters such as joint torque, movement smoothness, or EMG activity. Potential confounding factors, including spontaneous neurological recovery, individual variability in engagement, therapist-provided assistance, and cognitive or linguistic limitations, may also have influenced the observed outcomes. With respect to device-related considerations, although the cable-driven m-FLEX demonstrated good usability, its mechanical architecture still introduces restrictions, such as fixed joint-angle limiter increments and friction-related losses in Bowden cables, that may affect responsiveness, accuracy, and adaptability to different patient anatomies. Moreover, the system currently supports a limited set of ADL-like functional tasks, which may reduce the generalizability of training to more complex, multi-joint activities encountered in daily life. As a feasibility investigation, the present work provides preliminary evidence of acceptability and potential benefit but does not establish therapeutic effectiveness. Future research should involve larger randomized controlled trials, higher-intensity and adaptively parameterized training protocols, refined mechanical and control-system optimization (including friction modeling and EMG-based intention detection), and the incorporation of multimodal quantitative outcome measures to more accurately characterize motor recovery and the mechanisms underlying biomimetic robotic rehabilitation.

## 6. Conclusions

This pilot study evaluated the feasibility and therapeutic potential of a biomimetic, activities-of-daily-living (ADL)-based robotic rehabilitation protocol using a lightweight, cable-driven upper-limb exoskeleton (m-FLEX™) as an adjunct to conventional therapy for post-stroke patients. The incorporation of functional, goal-oriented tasks enabled participants to engage in training that more closely resembled everyday motor behaviors. Across the three cases, descriptive analyses indicated possible improvements in motor performance and functional independence, as reflected in Fugl-Meyer and Functional Independence Measure scores. Although the sample size was small (*n* = 3) and no control group was included, the findings suggest that early and intensive interventions informed by biomimetic principles may support neuroplastic processes and facilitate motor relearning. High levels of patient satisfaction also underscore the relevance of ergonomic design and client-centered approaches for promoting motivation and adherence. Future studies with larger samples, standardized protocols, and neurophysiological assessments are needed to confirm these preliminary observations and clarify the mechanisms of recovery. Even with these limitations, this study provides initial evidence that a biomimetic, ADL-based robotic rehabilitation approach is a feasible and potentially valuable strategy for supporting upper-limb functional recovery after stroke.

## 7. Patents

Dias, E. A. F., & Andrade, R. M. (2023) [25]. Órtese Robótica de Membro Superior Movida por Cabos de Aço para Reabilitação Neuromotora. BR Patent BR 20 2023 021372 9.

## Figures and Tables

**Table 1:** Maximum Range of Motion (RoM) and angular speed of the Upper-Limb Joints.

DoF	Max RoM [deg]	Max. Angular Speed [rad/s]
Flexion/Extension	120	6.0
Pronation/Supination	150	7.5
Hand	60	3.0

## Data Availability

The original contributions presented in this study are included in the article. Further inquiries can be directed to the corresponding author.

## References

[1] GBD 2021 Stroke Risk Factor Collaborators (2024). Global, regional, and national burden of stroke and its risk factors, 1990–2021: A systematic analysis for the Global Burden of Disease Study 2021. Lancet Neurol..

[2] World Health Organization (2021). Food Systems for Health: Information Brief.

[3] Smajlović D. (2015). Strokes in young adults: Epidemiology and prevention. Vasc. Health Risk Manag..

[4] Vargas I.M.P., Rodrigues L.P. (2022). Correlação entre espasticidade do membro superior e movimentação da mão no pós-AVC. Fisioter. Pesqui..

[5] Pollock A., Farmer S.E., Brady M.C., Langhorne P., Mead G.E., Mehrholz J., van Wijck F. (2014). Interventions for improving upper limb function a-er stroke. Cochrane Database Syst. Rev..

[6] Lopes F.M.R.F. (2021). Aspectos Éticos. Sistema Robótico Híbrido para Reabilitação de Membro Superior de Indivíduos Pós-Acidente Vascular Encefálico: Design Centrado no Usuário. Ph.D. Thesis.

[7] Duret C., Grosmaire A., Krebs H.I. (2019). Robot-Assisted Therapy in Upper Extremity Hemiparesis: Overview of an Evidence-Based Approach. Front. Neurol..

[8] Miao Q., Zhang M., Cao J., Xie S.Q. (2018). Reviewing high-level control techniques on robot-assisted upper-limb rehabilitation. Adv. Robot..

[9] Balasubramanian S., Garcia-Cossio E., Birbaumer N., Burdet E., Ramos Murguialday A. (2018). Is EMG a viable alternative to BCI for detecting movement intention in severe stroke?. IEEE Trans. Biomed. Eng..

[10] Duschau-Wicke A., Caprez A., Riener R. (2010). Patient-cooperative control increases active participation of individuals with sci during robot-aided gait training. J. Neuroeng. Rehabil..

[11] Han C.-H., Hwang H.-J., Lim J.-H., Im C.-H. (2018). Assessment of user voluntary engagement during neurorehabilitation using functional near-infrared spectroscopy: A preliminary study. J. Neuroeng. Rehabil..

[12] Grant T., Jolliffe L., Wales K., Schneider E., Drummond A.E., Lannin N.A. (2025). Activity-Based Interventions to Increase Independence After Stroke In the Hospital Setting: Protocol for a Systematic Review. JMIR Res. Protoc..

[13] Guidetti S., Eriksson G., von Koch L., Johansson U., Tham K. (2022). Activities in Daily Living: The development of a new client-centred ADL intervention for persons with stroke. Scand. J. Occup. Ther..

[14] Gull M.A., Bai S., Bak T. (2020). A review on design of upper limb exoskeletons. Robotics.

[15] Maciejasz P., Jörg E., Gerlach-Hahn K., Jansen-Troy A., Leonhardt S. (2014). A survey on robotic devices for upper limb rehabilitation. J. Neuroeng. Rehabil..

[16] Scibilia A., Prini A., Dinon T., Pedrocchi N., Caimmi M. Over three decades of upper-limb robotic neurorehabilitation: Drawing conclusions and future work. Proceedings of the 2024 IEEE 20th International Conference on Automation Science and Engineering (CASE).

[17] Lourenço B., Neto V., Andrade R.M. (2020). A Concept Design of an Adaptive Tendon Driven Mechanism for Active Soft Hand Orthosis. Proceedings.

[18] Silva R.C., Lourenco B.G., Ulhoa P.H., Dias E.A., da Cunha F.L., Tonetto C.P., Villani L.G., Vimieiro C.B., Lepski G.A., Monjardim M. (2023). Biomimetic design of a tendon-driven myoelectric soft hand exoskeleton for upper-limb rehabilitation. Biomimetics.

[19] Diaz F.H., Borrás Pinilla C., García Cena C.E. (2025). Exploring Robotic Technologies for Upper Limb Rehabilitation: Current Status and Future Directions. J. Sens. Actuator Netw..

[20] Guerrero-Mendez C.D., Blanco-Diaz C.F., Rivera-Flor H., Fabriz-Ulhoa P.H., Fragoso-Dias E.A., de Andrade R.M., Delisle-Rodriguez D., Bastos-Filho T.F. (2024). Influence of temporal and frequency selective patterns combined with csp layers on performance in exoskeleton-assisted motor imagery tasks. NeuroSci.

[21] Huo W., Mohammed S., Amirat Y., Kong K. (2018). Fast Gait Mode Detection and Assistive Torque Control of an Exoskeletal Robotic Orthosis for Walking Assistance. IEEE Trans. Robot..

[22] Dias E.A., Andrade R.M. (2020). Design of a cable-driven actuator for pronation and supination of the forearm to integrate an active arm orthosis. Proceedings.

[23] Dias E., Ulhoa P., Andrade R. (2023). Design of a 3 degree-of-freedom upper-limb active exoskeleton with cable-driven actuators for neuromotor rehabilitation. Proceedings of the 2023 IEEE Colombian Caribbean Conference (C3).

[24] Dias E., Ulhoa P., Milanezi R. (2024). EMG-based Co-Contraction Controller for an Upper-Limb Exoskeleton. Proceedings of the 2024 20th IEEE/ASME International Conference on Mechatronic and Embedded Systems and Applications (MESA).

[25] Dias E.A.F., Andrade R.M. (2023). Órtese Robótica de Membro Superior Movida por Cabos de Aço para Reabilitação Neuromotora.

[26] Lepski G., Milanezi R., Arevalo A. (2023). What Determines Success with Robot-Assisted Upper Limb Rehabilitation Following Stroke? A Systematic Review and Meta-Analysis. A Syst. Rev. Meta-Anal..

[27] Kwakkel G., Veerbeek J.M., van Wegen E.E.H., Wolf S.L. (2015). Constraint-induced movement therapy after stroke. Lancet Neurol..

[28] French B., Thomas L.H., Coupe J., McMahon N.E., Connell L., Harrison J., Sutton C.J., Tishkovskaya S., Watkins C.L. (2016). Repetitive task training for improving functional ability after stroke. Cochrane Database Syst. Rev..

[29] Kitago T., Krakauer J.W. (2013). Motor learning principles for neurorehabilitation. Handb. Clin. Neurol..

[30] Kwakkel G., Kollen B.J., Krebs H.I. (2008). Effects of robot-assisted therapy on upper limb recovery after stroke: A systematic review. Neurorehabilit. Neural Repair.

[31] Poli P., Morone G., Rosati G., Masiero S. (2013). Robotic technologies and rehabilitation: New tools for stroke patients’ therapy. BioMed Res. Int..

[32] He Y., Xu Y., Hai M., Feng Y., Liu P., Chen Z., Duan W. (2024). Exoskeleton-assisted rehabilitation and neuroplasticity in spinal cord injury. World Neurosurg..

[33] Cardone D., Perpetuini D., Di Nicola M., Merla A., Morone G., Ciancarelli I., Moretti A., Gimigliano F., Cichelli A., De Flaviis F. (2024). Robot-assisted upper limb therapy for personalized rehabilitation in children with cerebral palsy: A systematic review. Front. Neurol..

[34] Yan T., Cempini M., Oddo C.M., Vitiello N. (2015). Review of assistive strategies in powered lowerlimb orthoses and exoskeletons. Robot. Auton. Syst..

[35] Lázaro R., Vergara M., Morales A., Mollineda R.A. (2025). Multimodal Deep Learning Model for Cylindrical Grasp Prediction Using Surface Electromyography and Contextual Data During Reaching. Biomimetics.

[36] Lozano-Berrio V., Alcobendas-Maestro M., Perales-Gómez R., Pérez-Borrego Y., Gil-Agudo A., Polonio-López B., Cortés C., de los Reyes-Guzmán A. (2024). Can Robotic Therapy Improve Performance in Activities of Daily Living? A Randomized Controlled Trial in Sub-Acute Spinal Cord Injured Patients. Appl. Sci..

[37] Sharma P., Naglah A., Aslan S., Khalifa F., El-Baz A., Harkema S., D’Amico J. (2023). Preservation of functional descending input to paralyzed upper extremity muscles in motor complete cervical spinal cord injury. Clin. Neurophysiol..

[38] Cacho E.W.A., de Melo F.R.L.V., de Oliveira R. (2004). Avaliação da recuperação motora de pacientes hemiplégicos através do protocolo de desempenho físico Fugl-Meyer. Rev. Neurociênc..

[39] Niessen M., Janssen T., Meskers C., Koppe P., Konijnenbelt M., Veeger D. (2008). Kinematics of the contralateral and ipsilateral shoulder: A possible relationship with post-stroke shoulder pain. J. Rehabil. Med..

[40] Bolognini N., Russo C., Edwards D.J. (2016). The sensory side of post-stroke motor rehabilitation. Restor. Neurol. Neurosci..

[41] Smania N., Montagnana B., Faccioli S. (2003). Rehabilitation of somatic and related deficit of motor control in patients with pure sensory stroke. Arch. Phys. Med. Rehabil..

[42] Vianna F.P., de Lorenzo A.P.C., Oliveira É.F., Resende S.M. (2008). Medida de independência funcional nas atividades de vida diária em idosos com sequelas de acidente vascular encefálico no Complexo Gerontológico Sagrada Família de Goiânia. Rev. Bras. Geriatr. Gerontol..

[43] Rissetti J., Feistauer J.B., Luiz J.M., Silveira L.S., Ovando A.C. (2020). Independência funcional e comprometimento motor em indivíduos pós-ave da comunidade. Acta Fisiatr..

[44] Zeiler S., Krakauer J. (2013). The interaction between training and plasticity in the poststroke brain. Curr. Opin. Neurol..

[45] Kennedy J., Davis J. (2017). Clarifying the construct of occupational engagement for occupational therapy practice. Occup. Ther. J. Res..

[46] Manna S., Dubey V. (2018). Comparative study of actuation systems for portable upper limb exoskeletons. Med. Eng. Phys..

